# Foreign language comprehension achievement: insights from the cognate facilitation effect

**DOI:** 10.3389/fpsyg.2015.00588

**Published:** 2015-05-06

**Authors:** Aina Casaponsa, Eneko Antón, Alejandro Pérez, Jon A. Duñabeitia

**Affiliations:** Basque Center on Cognition, Brain and LanguageDonostia, Spain

**Keywords:** cognate effect, reading comprehension achievement, second language learning, formal L2 lessons, lexical decision, foreign language acquisition

## Abstract

Numerous studies have shown that the native language influences foreign word recognition and that this influence is modulated by the proficiency in the non-native language. Here we explored how the degree of reliance on cross-language similarity (as measured by the cognate facilitation effect) together with other domain-general cognitive factors contribute to reading comprehension achievement in a non-native language at different stages of the learning process. We tested two groups of native speakers of Spanish learning English at elementary and intermediate levels in an academic context. A regression model approach showed that domain-general cognitive skills are good predictors of second language reading achievement independently of the level of proficiency. Critically, we found that individual differences in the degree of reliance on the native language predicted foreign language reading achievement, showing a markedly different pattern between proficiency groups. At lower levels of proficiency the cognate facilitation effect was positively related with reading achievement, while this relation became negative at intermediate levels of foreign language learning. We conclude that the link between native- and foreign-language lexical representations helps participants at initial stages of the learning process, whereas it is no longer the case at intermediate levels of proficiency, when reliance on cross-language similarity is inversely related to successful non-native reading achievement. Thus, at intermediate levels of proficiency strong and direct mappings from the non-native lexical forms to semantic concepts are needed to achieve good non-native reading comprehension, in line with the premises of current models of bilingual lexico-semantic organization.

## Introduction

Nowadays, most countries include a second language in their educational curricula, and English has been, so far, the most frequently taught non-native language in formal academic contexts. The generalized increase in second language learning has stimulated a lot of research into the cognitive mechanisms underlying non-native language learning and the way in which newly acquired sounds, words, and grammatical structures from the non-native language interact with preexisting representations from the native language. In the current study we explored how individual differences in the degree of implicit reliance on cross-language similarity (i.e., the spontaneous sensitivity to orthographic, phonological and semantic similarities between words from different languages) observed at early stages of the learning process contribute to reading comprehension achievement in a non-native language together with other domain-general cognitive factors.

There is extensive empirical evidence showing that word recognition in a foreign language is influenced by the native language (van Heuven et al., 1998; Dijkstra and van Heuven, 2002; Kroll and Dijkstra, 2002; Kroll et al., 2002; Lemhöfer and Dijkstra, 2004; among others) and that the degree of reliance on the native language depends upon second language proficiency. In line with this idea, the Revised Hierarchical Model (RHM) of bilingual lexico-semantic organization (Kroll and Stewart, 1994; Kroll et al., 2010) predicts that semantic access during reading comprehension is mediated by inter-lingual links at lower levels of proficiency in the non-native language (L2) and that mediation through the native language (L1) is necessary to achieve full access to conceptual representations while reading in the L2. Critically, the same theoretical account also implies that direct links between L2 lexical representations and language-independent semantic representations are created at higher levels of the L2 proficiency, such that reliance on the L1 during L2 reading diminishes as a function of increased L2 proficiency. The RHM is a developmental model of L2 acquisition initially proposed to account for performance in translation production. However, its predictions also fit the changes observed in L2 word recognition (see Kroll et al., 2010, for a summary; but see also Brysbaert and Duyck, 2010). One of the main assumptions of the RHM that it is still a matter of debate is the existence of separate lexicons, an assumption mostly incompatible with neural and computational models of bilingualism. Other models have been put forward to overcome this issue, and the Developmental Bilingual Interactive-Activation model (BIA-d; Grainger et al., 2010) appears to lead the way as an alternative theoretical framework. The BIA-d is a dynamic model of L2 learning that combines the main features of the developmental changes in L2 acquisition proposed by the RHM and the interactive-activation principles of the Bilingual Interactive Activation model that assumes a unified mental lexicon (BIA-model; first described by Grainger and Dijkstra, 1992, and implemented by van Heuven et al., 1998).

According to the BIA-d, the acquisition of L2 words requires direct mappings with their translation equivalents in the native language, direct mappings with language-independent semantic representations, and information regarding the corresponding language tag, which, in turn, is connected to L1 word forms. These connections follow interactive-activation principles and their strength depends on the proficiency in the L2. The BIA-d predicts that at higher levels of L2 proficiency, the weights of excitatory connections between L2 lexical representations and language-independent semantic representations increase, while the strength of links between L2 and L1 word forms (i.e., L1 mediation) decreases. Another key aspect of this model is that the decrease of reliance on the L1 is modulated by inhibitory connections from the L2 language tag to the L1 word forms. Thus, as reliance on L1 translation during L2 word comprehension decreases as a function of increased proficiency, the inhibitory connections between L2 and L1 words increase. This is a critical aspect of this model since it features L2 word forms integrated into a common lexicon, thus allowing for inhibitory effects to emerge for both L2 and L1 lexical representations. According to this model, newly acquired L2 words are connected to their translation equivalents and to their corresponding semantic representations via excitatory connections. These connections are then strengthened as a function of increased exposure to these words. At higher levels of proficiency, and with the integration of the L2 words into the L1 lexicon, the strength of the connections between L2 words and their L1 translations is reduced due to the development of inhibitory connections. At this stage, L1 and L2 lexical representations would be automatically activated during reading (i.e., there would be language-independent lexical access to a single multilingual lexicon), and lexical competition would be modulated by the relative resting activation threshold of each word form as well as formal overlap and lexical distance between L1 and L2 representations.

Both the RHM and BIA-d models predict similar effects during L2 reading at early stages of L2 learning, since the acquisition and processing of L2 words is supposedly mediated by L1 translations during these early stages. Both the models also suggest that reliance on L1 translations decreases as a function of increased L2 proficiency, given the strengthening of the direct links between L2 lexical forms and corresponding semantic representations. However, at higher and native-like levels of proficiency the models differ in the way L2 and L1 lexical items are represented (i.e., in two different lexicons in the RHM and in a single lexicon in the BIA-d). Thus, even though the two models account for the development of L2 acquisition, they differ substantially as regards predictions on the degree of cross-language interactions (excitatory and inhibitory) at different levels of word processing.

Reliance on cross-language similarity is a critical factor that has been shown to modulate not only L2 lexical access, but also L2 word learning. Research on non-native word learning has shown that new foreign words that follow the phonotactic or orthotactic rules of the native language elicit stronger and earlier behavioral and neural changes than new words that are at odds with the native language phonotactic or orthotactic rules (e.g., Mestres-Missé et al., 2007; Borovsky et al., 2010, 2012). In a nutshell, studies exploring native and non-native vocabulary acquisition show that the lexico-semantic representations of newly acquired vocabulary are better established when these representations overlap with the native language at form-based linguistic levels (orthography and phonology), thus suggesting that non-native cognate words (i.e., translation equivalents with overlapping orthographic and/or phonological representations; e.g., *guitar* for a native Spanish speaker learning English, translated as *guitarra* in Spanish) are easier to learn and integrate in the lexicon than non-cognate words (i.e., translation equivalents without ortho-phonological overlap; e.g., the English word *house*, translated as *casa* in Spanish; see Ellis and Beaton, 1993; Kroll et al., 1998; Lotto and De Groot, 1998; De Groot and Keijzer, 2000; De Groot and van Hell, 2005).

In order to better characterize the impact of cross-language similarity during L2 reading, Lemhöfer et al. (2008) investigated how various non-native speakers of English responded to a large set of English words. They tested Dutch, French, and German learners of English in a progressive demasking task in which participants had to identify visually presented words in their L2 (English), and tested the modulation of word identification time by several within and between-language factors. They found that the cognate status of words was the best cross-language performance predictor in all the groups tested. Indeed, cognate words were easier to recognize than non-cognate words in all three groups of bilinguals reading in English. The cognate effect is thus pervasive across languages and probably generalizable.

The fact that non-native cognate words are recognized and produced faster and more accurately than non-native non-cognate words (e.g., *guitarra-guitar* vs. *casa-house*) is a well-documented finding (see Caramazza and Brones, 1979; Cristoffanini et al., 1986; De Groot and Nas, 1991; Sánchez-Casas et al., 1992; Dijkstra et al., 1998, 1999; Font, 2001; Van Hell and Dijkstra, 2002; Kroll et al., 2002; Lemhöfer and Dijkstra, 2004; Lemhöfer et al., 2004; Voga and Grainger, 2007; Davis et al., 2010; Duñabeitia et al., 2010; Midgley et al., 2011; Peeters et al., 2013, among many others). Critically, the cognate facilitation effect has also been shown to decrease as a function of proficiency in the non-native language (e.g., Bultena et al., 2014). The magnitude of the cognate effect is larger at lower levels of second language proficiency, suggesting a greater reliance on preexisting native-language representations (Potter, 1979; Kroll and Stewart, 1994; Kroll and De Groot, 1997; Dijkstra and van Heuven, 2002; Kroll and Dijkstra, 2002; Kroll et al., 2002; Grainger et al., 2010). In contrast, at higher levels of second language proficiency, the facilitative effects of cognate words compared to non-cognate words are reduced, possibly indicating lower reliance on native-language representations (e.g., Potter et al., 1984; Kroll et al., 2010), and the potential inclusion of L2 words in an integrated lexicon in which excitatory and inhibitory connections between L2 and L1 representations play a critical role (Grainger et al., 2010).

In the current study we set out to explore whether the cognate effect, a well-known psycholinguistic effect associated with cross-language interactions, contributes to non-native reading comprehension achievement in a formal academic context. Specifically, we tested whether the cognate effect measured during the learning process can predict non-native language achievement at the end of the academic year. Furthermore, the current study tried to better characterize how the degree of reliance on cross-language similarity (as measured by the cognate effect) at different levels of the learning process contributes to reading comprehension achievement.

Considering that preceding studies have consistently shown that the cognate effect is a highly reliable psycholinguistic measure, and considering the evidence suggesting that the magnitude of the cognate effect decreases as a function of increased proficiency in the non-native language, in the current study we explored whether the cognate effect is a reliable measure able to explain individual differences in second language learning. Consistent with the literature reviewed above, we expected to find a substantial cognate facilitation effect at initial stages of formal second language learning and a reduction of this effect at higher proficiency levels. Moreover, given that at initial stages of second language learning readers tend to rely on their L1 in order to consolidate and integrate newly acquired words (Kroll and Stewart, 1994; Dijkstra and van Heuven, 2002; Kroll et al., 2002, 2010; Grainger et al., 2010) reliance on cross-language similarity was expected to positively contribute to L2 reading comprehension in novice readers. We thus predicted that the magnitude of the cognate effects would relate positively to general non-native reading comprehension achievement in novice readers of L2. Conversely, we predicted that reliance on cross-language similarity (as measured by the cognate effect) would relate negatively to general L2 reading comprehension achievement at higher levels of proficiency. To the best of our knowledge, this is the first study aimed at exploring how cross-language reliance modulates general reading comprehension at different stages of the L2 academic learning process.

We tested two groups of participants attending to two different levels of English learning according to the Common European Framework of Reference for Languages: Learning, Teaching, Assessment (CEFR). The low proficiency group was enrolled at the A2 level and the intermediate proficiency group was enrolled at the B1 level of the CEFR. All participants were tested (independently of their level) at the beginning of the second semester with a battery including various psychological and psycholinguistic measures. We then explored the contribution of these indices to the final scores in the official reading comprehension assessment completed at the end of the academic year. The reading comprehension tests followed the CEFR guidelines for each level and were evaluated by qualified professors from the Public Language School at the end of the scholar term following CEFR standards.

## Experiment 1: Low Proficient Learners of English (Level A2)

### Methods

#### Participants

Sixty two native Spanish speakers (45 Females) attending English lessons (level A2 of the CEFR) at the Public Language School in Donostia took part in the experiment. Their mean age was 43.16 years (range: 19–68; SD: 12.44). Participants’ self-ratings of proficiency in English are listed in **Table 1**. None of them reported history of neuropsychological disorder and all had normal or corrected-to-normal vision. All participants gave their written informed consent in accordance with guidelines approved by the Ethics and Research Committees of the Basque Center on Cognition, Brain and Language. The study was also performed in accordance with the ethical standards set in the Declaration of Helsinki.

**Table 1:** Means (and SD) of participants language report in Experiment 1.

Item	Mean (SD)
Age of English acquisition	25.56 (18.37)
**Self-ratings**
English Spoken	3.11 (1.64)
English Written	3.53 (1.59)
English Understand	3.53 (2.04)
General level of English	3.52 (1.55)
General level of Spanish	8.81 (0.87)

*Self-ratings were given on a scale from 1 (low) to 10 (high)*.

#### Materials and Procedure

The initial assessment of the participants was carried out at the end of the first school term (February), 4 months before they were tested in the official exams of the Public Language School (June). Every participant completed a battery consisting of three tasks during class time. They volunteered to leave their regular classes in groups of 10 for a 30-min session held in the multimedia room of the Public Language School. After receiving basic information about the aims of the study and the tasks they had to complete, participants completed a series of questionnaires on their linguistic and socio-demographic background. The three main tasks that participants completed were (1) an English lexical decision task (used to estimate students’ cognate effect; see **Table 2** and below), (2) a working memory test using the forward number retention task from the Wechsler Adult Intelligence Scale-Fourth Edition (WAIS-IV; Wechsler, 2008), and (3) an abridged version of the Kaufman Brief Intelligence Test (KBIT; Kaufman, 1990; Kaufman and Kaufman, 2004) to have an estimate measure of their non-verbal intelligence. The working memory test (WAIS-IV) included eight sequences of numbers read aloud by the experimenters at the pace of one digit per second. Participants had to write the digits in the same order they heard them once they received the command from the experimenters (right after giving the last digit of each sequence). The number of consecutive correct responses was used as a rating of working memory (WM). The sequences followed a progressively increasing level of difficulty, ranging from 2 to 9 digits per sequence. Non-verbal IQ was assessed with an abridged version of the matrices subtest from the KBIT test. Participants had to respond to as many matrices as they could during a 6-min interval. The total amount of correct answers during this period was used as a measure of fluid intelligence. The results of all tasks included in the experimental session are summarized in **Table 3**.

**Table 2:** Mean values and SD for the stimuli used in the experiment.

	Frequency	Length	Number of neighbors	Imageability
Cognates	56.04 (63.08)	6.71 (1.67)	1.41 (2.87)	4.50 (1.1)
Non-cognates	54.06 (54.27)	6.66 (1.40)	1.69 (2.63)	4.63 (1.07)

**Table 3:** Scores obtained for the tasks in Experiment 1 and scores obtained at the end of the learning process for A2 group of English learners.

Task	Mean (SD)
Cognate effect (in ms)	60.94 (80.26)
Cognate effect (% of errors)	16.70 (10.76)
WM estimate	4.22 (0.88)
IQ estimate	18.99 (4.03)
Reading comprehension	16.19 (3.05)

*Reading comprehension scores ranged from 0 to 20 assessed using the criteria set on by the CEFR*.

A set of 200 English nouns were selected from the N-Watch database (Davis and Perea, 2005) for the lexical decision task. Half of the words were Spanish-English cognates (e.g., MINUTE, *minuto* in Spanish), and the other half were non-cognates (e.g., SUMMER, *verano* in Spanish). Orthographic overlap was assessed following the same methodology used in Duñabeitia et al. (2013; see also, Schepens et al., 2011). According to a 0-to-1 continuum of cognate status^1^ (with higher values corresponding to greater overlap across languages), cognate words ranged from 0.7 to 1 (mean = 0.82, SD = 0.09) and non-cognate words ranged from 0 to 0.3 (mean = 0.11, SD = 0.10). Cognate and non-cognate words were matched for frequency, length, number of orthographic neighbors and imageability (see **Table 2**). A set of 200 non-words following English orthotactic rules (e.g., ENCHORY) was generated using Wuggy (Keuleers and Brysbaert, 2010). Each trial consisted of the presentation of a fixation cross (“+”) in the middle of the screen for 500 ms, immediately followed by the presentation of a letter string that could be either a real English word or not. Participants were instructed to press the “L” button of the keyboard for real words and the “S” button for non-words. Letter strings remained on the screen for 2500 ms or until a response was given. Prior to the presentation of the experimental trials in a random order generated for each participant, six practice trials were presented in order to familiarize participants with the task. The lexical decision experiment lasted for ∼10 min and was created using Experiment Builder.

Results from questionnaires and from the three experimental tests were then used to predict participants’ outcome in the official assessment for reading comprehension skills by the Public Language School following the standard methods used in Common European Framework of Reference for Languages (2011^2^) at the end of the academic year (4 months after completion of the experimental session). Grades in the official examination ranged from 0 to 20, 12 being the minimum score required to obtain a pass for the A2 level. The criteria used to assess reading comprehension included the understanding of short, simple texts on familiar matters of a concrete type which consist of high frequency everyday or job-related language. Furthermore, it also included the understanding of basic types of standard routine letters and faxes (enquiries, orders, letters of confirmation, etc.) on familiar topics, the understanding of short simple personal letters, and the understanding of everyday signs and notices.

### Results and Discussion

First, trials from the lexical decision task associated with erroneous responses and responses latencies that were above or below 2 SD from the participant-based means in each condition were excluded from the RT analysis (5.67% of the data). Second, ANOVAs on the RTs and percentage of errors were conducted in order to test for an overall cognate effect in the test group (i.e., comparing latencies and accuracy data between cognate and non-cognate words). Third, a regression analysis was carried out using participants’ scores in reading comprehension at the end of the academic year as the dependent variable, together with the following list of predictor variables: the cognate effect (in ms; obtained by subtracting RTs to cognate words from RTs to non-cognate words), the general self-rating of English proficiency (on a scale from 1 to 10), participants’ age of acquisition of English and their chronological age (both in years), and the results from the WM and IQ tests (raw scores).

**FIGURE 1:**
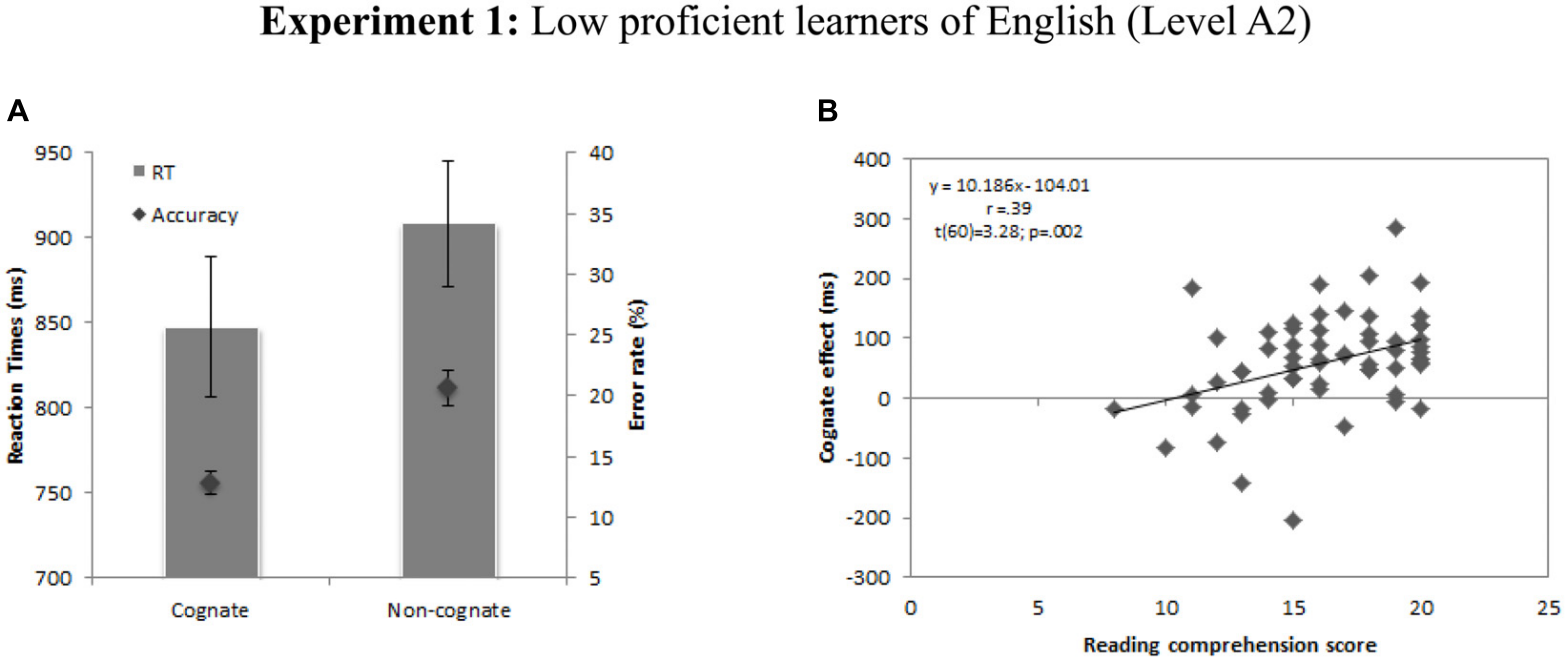
**(A)** Mean latencies and error rates for cognate and non-cognate words in Experiment 1. Error bars represent 95% confidence intervals. **(B)** Correlation between individuals’ cognate effects and reading comprehension scores for A2 group of English learners.

ANOVAs aimed at confirming the existence of a cognate effect in the lexical decision task showed a significant main effect of Cognate status on RTs [*F*1(1,61) = 35.74, *p* < 0.001; *F*2(1,99) = 7671.90, *p* < 0.001] and accuracy [*F*1(1,61) = 40.83, *p* < 0.001; *F*2(1,99) = 72.97, *p* < 0.001], such that cognate words were recognized faster and more accurately than non-cognate words (see **Figure 1A**).

Next, the mean cognate effect was entered into a regression model (backward multiple regression) together with the age of the participants, their self-perceived English level, their age of acquisition of English, and their scores in the WM and IQ tests in order to investigate the relative contribution of each factor for the final scores obtained in the official examinations from the Public Language School. The simplest model accounting for most of the variance included three out of the six initial predictors and was reached in three steps. Age of acquisition (as measured by age of first contact with English) was first dropped from the most complex model without losing explanatory capacity [βs = 0.11, *t*(61) = 0.68, *p* = 0.49], then the chronological age of the participants was also dropped from the model in a later step [βs = 0.15, *t*(61) = 1.11, *p* = 0.27] and finally the WM was also dropped from the final model [βs = 0.15, *t*(61) = 1.26, *p* = 0.21; see **Table 4**].

**Table 4:** Model Summary for the backward multiple regression analysis with reading comprehension as a dependent variable in Experiment 1.

Model	*R*	*R*^2^	Adjusted *R*^2^	Change statistics		
				*R*^2^ change	*F* change	*p* change
1	0.57^a^	0.32	0.25	0.32	4.29	0.01
2	0.56^b^	0.31	0.25	-0.01	0.46	0.50
3	0.55^c^	0.30	0.25	-0.02	1.24	0.27
4	0.53^d^	0.28	0.24	-0.02	1.59	0.21

*^a^Predictors: cognate effect, WM estimate, IQ estimate, level English, age, AoA English*.

*^b^Predictors: cognate effect, WM estimate, IQ estimate, level English, age*.

*^c^Predictors: cognate effect, WM estimate, IQ estimate, level English*.

*^d^Predictors: cognate effect, IQ estimate, level English*.

The model was statistically significant [*F*(4,58) = 7.46, *p* < 0.001] and accounted for approximately 25% of the variance of reading comprehension (*R*^2^ = 0.28, Adjusted *R*^2^ = 0.24). The resulting regression weights, *t* values, significance levels, structured coefficients, squared partial correlations and their correlations with reading comprehension are summarized in **Table 5**. Higher scores in reading comprehension as assessed by official examinations were significantly predicted by higher cognate effects, higher levels of self-rating perception of English proficiency, and higher scores in IQ. β-weights revealed that all predictors received similar credit in the regression equation (see **Table 5**), and a closer inspection of the structure coefficients suggested that cognate effect contributed most to the variance explained (*R*^2^) with the largest absolute value for both the β-value and the structure coefficient (β = 0.36, *r*_s_ = 0.74, r_s_^2^ = 54%), followed by IQ (β = 0.25, *r*_s_ = 0.60, r_s_^2^= 36%) and self-rating of perceived proficiency (β = .28, r_s_= 0.18, r_s_^2^ = 11%). Interestingly, the cognate effect measured at the end of the first term turned out to be a good predictor of future reading comprehension level at the end of the academic year. That is, non-native language reading achievement is highly influenced by the magnitude of the cognate effect at initial stages of the learning process.

**Table 5:** Results of the multiple regression model using the backward method in Experiment 1.

Predictor	β	*t*	*p*	*r*	sr^2^	*r*_s_	r_s_^2^
Cognate effect	0.36	3.30	0.002	0.39	0.14	0.74	0.54
Self-rating of English proficiency	0.28	2.45	0.02	0.18	0.07	0.34	0.11
IQ estimate	0.25	2.17	0.03	0.32	0.06	0.60	0.36

*r, Pearson correlation; sr^2^, squared semi-partial correlation; *r*_*s*_, structure coefficient = r/R. *r*_*s*_^2^, squared structure coefficient = r^2^∖R^2^*.

## Experiment 2: Intermediate proficient learners of English (Level B1)

### Methods

#### Participants

One hundred and five native Spanish speakers (65 Females) took part in this study. All of them were attending English lessons (level B1 of CEFR) at the Public Language School in Donostia, Spain. Their mean age was 38.76 (range: 19–69; SD: 13.05). Participants’ self-ratings of proficiency in English are listed in **Table 6**. None of them reported a history of neuropsychological disorder and all had normal or corrected-to-normal vision.

**Table 6:** Mean (and SD) of participants language report.

Item	Mean (SD)
Age of English acquisition	19.90 (16.30)
**Self-ratings**
English Spoken	4.15 (1.32)
English Written	4.93 (1.47)
English Understand	4.44 (1.62)
General level of English	4.67 (1.29)
General level of Spanish

Self-ratings were given on a scale from 1 (low) to 10 (high).

#### Materials and Procedure

These were the same as in Experiment 1. The criteria used to assess reading comprehension for the B1 level included the understanding of texts of high frequency everyday or job-related language, and the understanding of events, feelings and whishes in personal letters. Furthermore, it also included the understanding of longer texts requiring students to be able to locate and gather desired information from different parts of a text, as well as to recognize significant aspects in straightforward newspaper articles on familiar subjects.

### Results and Discussion

Data analyses were carried out following the same criteria as in Experiment 1. Trials associated with erroneous responses and responses latencies that were above or below 2 SD from the participant-based means in each condition were excluded from the RT analysis (2.99% of the data). The same factors used in Experiment 1 were included in a backward multiple regression model using the final scores of reading comprehension as a dependent variable. Descriptive results can be found in **Table 7**.

**Table 7:** Scores obtained for the tasks in Experiment 2 and scores obtained at the end of the learning process for B1 group of English learners.

Task	Mean (SD)
Cognate Effect (in ms)	47.45 (48.43)
Cognate Effect (% of errors)	10.12 (6.84)
WM Estimate	4.65 (1.03)
IQ Estimate	20.55 (3.76)
Reading Comprehension	15.32 (3.35)

*Reading comprehension ranged from 0 to 20 assessed using the criteria set on by the CEFR*.

**FIGURE 2:**
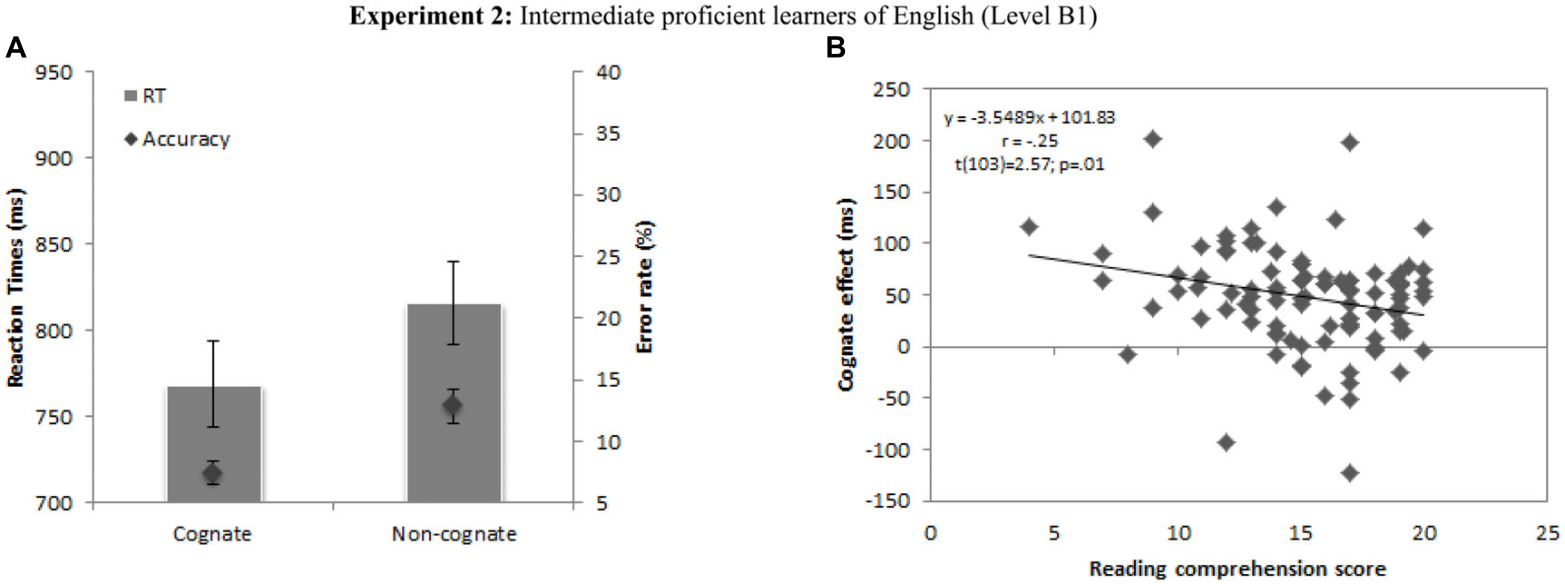
** (A)** Mean latencies and error rates for cognate and non-cognate words in Experiment 2. Error bars represent 95% confidence intervals. **(B)** Correlation between individuals’ cognate effects and reading comprehension scores for B1 group of English learners.

Analyses of variance (ANOVAs) aimed at confirming the existence of a cognate effect in the lexical decision task showed a significant main effect of Cognate status in the RT data [*F*1(1,104) = 100.78, *p* < 0.001; *F*2(1,99) = 4896.89, *p* < 0.001] and in the accuracy data [*F*1(1,104) = 106.17, *p* < 0.001; *F*2(1,99) = 99.35, *p* < 0.001], demonstrating that cognate words were recognized faster and more accurately than non-cognate words (see **Figure 2A**).

The mean cognate effect along with the same predictors used in Experiment 1 were entered into a backward multiple regression model with the final scores obtained in the official examinations from the Public Language School as a dependent variable (see **Table 8**). The simplest model accounting for most of the variance included four out of the six initial predictors and was reached in three steps. Self-perceived English proficiency was first dropped from the most complex model without losing explanatory capacity [βs < 0.005, *t*(104) = -0.03, *p* > 0.95], and the chronological age of the participants was also dropped from the model in a later step (βs < 0.07, *t*(104) = 0.45, *p* > 0.65; see **Table 9**).

**Table 8:** Model summary for the backward multiple regression analysis with written comprehension as a dependent variable.

Model	*R*	*R*^2^	Adjusted *R*^2^	Change statistics		


				*R*^2^ Change	*F* change	*P* change
1	0.47^a^	0.22	0.17	0.22	4.51	0.01
2	0.47^b^	0.22	0.18	0.01	0.01	0.98
3	0.47^c^	0.22	0.18	-0.01	0.20	0.67

*^a^Predictors: cognate effect, WM estimate, IQ estimate, AoA English, age, level English*.

*^b^Predictors: cognate effect, WM estimate, IQ estimate, AoA English, age*.

*^c^Predictors: cognate effect, WM estimate, IQ estimate, AoA English*.

**Table 9:** Results of the multiple regression model using backward method.

Predictor	β	*t*	*p*	*r*	sr^2^	*r*_s_	r_s_^2^
Cognate effect	-0.20	-2.25	0.03	-0.25	0.04	0.53	0.28
Age of acquisition	-0.19	-1.87	0.06	-0.28	0.04	0.61	0.36
IQ estimate	0.22	2.24	0.03	0.34	0.04	0.73	0.53
WM estimate	0.21	2.34	0.02	0.17	0.03	0.36	0.13

*r, Pearson correlation; sr^2^, squared semi-partial correlation; *r*_*s*_ = structure coeficient = r/R. *r*_s_^2^, squared structure coefficient = *r*^2^∖*R*^2.^*

The model was statistically significant [*F*(4,104) = 6.80, *p* < 0.001] and accounted for ∼20% of the variance of reading comprehension (*R*^2^ = 0.22, Adjusted *R*^2^ = 0.18). The resulting regression weights, *t* values, significance levels, structured coefficients, squared partial correlations and their correlations with reading comprehension are summarized in **Table 5**. Higher scores in reading comprehension as assessed by official examinations were significantly predicted by lower cognate effects, lower age of acquisition of English, and higher WM and IQ. β-weights revealed that all predictors received similar credit in the regression equation (see **Table 5**), and a closer inspection of the structure coefficients suggested that IQ contributed most to the variance explained (*R*^2^) with the largest absolute value for both the β-value and the structure coefficient (β = 0.22, *r*_s_ = 0.73, r_s_^2^ = 54%), followed by the cognate effect and age of acquisition (β = 0.20, *r*_s_ = 0.53, r_s_^2^ = 28% and β = 0.19, *r*_s_ = 0.61, r_s_^2^ = 37%) and WM (β = 0.21, *r*_s_ = 0.36, r_s_^2^ = 13%). Interestingly for the purposes of the current study, the cognate effect measured at the end of the first term turns out to be a good predictor of future reading comprehension level at the end of the academic year. That is, non-native language reading achievement was highly influenced by the magnitude of the cognate effect at earlier stages of the learning process. However, the direction of this effect was the opposite of that found in Experiment 1. In Experiment 1 larger cognate effects significantly predicted better reading achievement, but in Experiment 2 the magnitude of these effects were negatively related to reading comprehension achievement, so that learners with smaller cognate effects scored better in official examinations (see **Figure 2B**). Moreover, the cognate effect was not correlated with any other general performance factor such as participants’ scores in the WM and IQ tests (both *r*_s_ < 0.15; *p*_s_ > 0.05), suggesting a neat link between cognate effect and reading comprehension achievement.

## General Discussion

The main goal of the present study was to investigate relations between the cognate effect and non-native language acquisition and to explore the extent to which reliance on cross-language similarity contributes to further reading achievement in a formal academic context at different levels of proficiency. We aimed at characterizing how the degree of reliance on cross-language similarity contributes to reading comprehension achievement at different levels of L2 proficiency. To do so, we first tested the cognate effect in two groups of native Spanish speakers who were learning English at a Public Language School, corresponding to the levels A2 (low-proficiency level) and B1 (intermediate-proficiency level) of CEFR. Participants were also assessed for WM and fluid intelligence, as domain-general cognitive factors have already been proven to have an effect on reading acquisition (Papagno and Vallar, 1995; Kempe et al., 2010; Lopez-Barroso et al., 2011). We then investigated the contribution of these factors (together with other socio-demographic and linguistic factors) on the final reading comprehension scores obtained 4 months afterward at the end of the school year (assessed using the criteria set on by the CEFR).

As expected, we found a significant cognate effect in a single-word presentation lexical decision task in the non-native language, showing that cognate words were processed faster and more accurately than non-cognate words in both our participant groups^3^, in line with previous literature (Dijkstra et al., 1998, 1999, 2010; Font, 2001; Van Hell and Dijkstra, 2002; Lemhöfer and Dijkstra, 2004; Lemhöfer et al., 2004; Duñabeitia et al., 2010; Midgley et al., 2011; Peeters et al., 2013).

These results are in line with the RHM (Kroll and Stewart, 1994; Kroll et al., 2010) that suggests that semantic access for L2 words is highly mediated by the activation of their corresponding L1 translation equivalents. As a consequence, L2 words that highly overlap with their L1 translations at the orthographic level (i.e., cognates) would be recognized faster and more accurately than non-cognates, since L1-mediation would be facilitated as a consequence of the high similarity. Furthermore, according to the RHM, a substantial increase of L2 proficiency would boost the creation and strengthening of direct links from L2 words to their corresponding semantic representations, yielding reduction of the strength of pre-existing links between L2 and L1 lexical representations. Within this framework, greater cognate effects are expected for lower proficiency stages of L2 learning due to the greater involvement of L1 mediation.

In a similar vein, the different cognate effects reported for the two groups of participants fit well with the BIA-d model (Grainger et al., 2010), which also predicts faster reaction times and greater accuracy rates for cognate words over non-cognates given that the orthographic, lexical and semantic representations activated by L2 cognate words overlap with those from their L1 translation equivalents. Besides, this model suggests that cognate words also present higher relative frequency of use than non-cognates, thus speeding up their recognition. At low levels of L2 proficiency the BIA-d predicts similar results than those predicted by the RHM. Newly acquired L2 words highly rely on interconnections with their L1 translations as well as with the corresponding semantic representations, leading to greater cognate effects than at higher levels of L2 proficiency. However, when the L2 proficiency increases and L2 words are integrated into the L1 lexicon (note that the BIA-d model assumes a single integrated lexicon), the interconnections with their L1 translations decrease as a result of the inhibition coming from the L2 language nodes, and hence the diminished cognate effects at higher levels of L2 proficiency. Therefore, the BIA-d model correctly accounts for the different magnitudes of cognate effects observed in the B1 and A2 groups. While the theoretical source of the cognate effects is not a critical issue under debate in the current study, we acknowledge that both theoretical frameworks (i.e., RHM and BIA-d model) can account for the reported differences in the cognate effects.

More importantly and directly related to the issue at stake in the current study, the results from the multiple regression analyses show that the magnitude of the cognate effect measured at the end of the first term is a reliable predictor of non-native reading comprehension achievement at the end of the academic year for both groups of second language learners. Interestingly, in Experiment 1 we found that individual differences in the cognate effect were positively related with the reading comprehension scores at the end of the academic year at lower proficiency levels (A2). That is, those L2 learners who showed larger cognate facilitation effects at the end of the first academic term (February) obtained higher reading comprehension scores at the end of the academic year. These results suggest a positive relationship between the degree of reliance on cross-language similarity (as measured by the cognate effect) and foreign reading comprehension achievement at low levels of formal non-native language learning. In contrast, in Experiment 2 we found that individual differences in the magnitude of the cognate effect were negatively related with the final scores in the reading comprehension assessment at intermediate proficiency levels (B1). Those intermediate L2 learners who achieved better reading comprehension scores at the end of the academic year were the ones who relied less on cross-language similarities when reading words in L2 at the end of the first academic term.

More specifically, results from the multiple regression model in the low-proficiency group (Experiment 1) showed that final reading comprehension scores were best explained by higher cognate effects, higher self-ratings of self-perceived proficiency, and higher scores in the non-verbal intelligence test. The model explained ∼25% of the variance in reading comprehension and included only the three factors mentioned above. In contrast, the resulting regression model for the intermediate-proficiency group (Experiment 2), which approximately explained 20% of the variance in reading comprehension scores, included four variables as significant predictors: the cognate effect, participants’ age of acquisition of the non-native language, WM skills and an estimate of non-verbal intelligence. Results from this group showed that better reading comprehension achievement was primarily predicted by lower cognate effects (i.e., smaller RT differences between the recognition of cognates and non-cognates) and by lower age of non-native language acquisition (i.e., earlier contact with English as a non-native language), and by higher scores in the WM and non-verbal intelligence tests.

Taken together the results from both groups of participants suggest that the link between native- and foreign-language lexical representations helps participants at initial stages of the learning process, while this is not the case at intermediate levels of proficiency. Thus, at intermediate levels of proficiency strong and direct mappings from the non-native lexical forms to semantic concepts seem needed to achieve good non-native reading comprehension scores. These results are in line with the predictions of both the RHM (Kroll and Stewart, 1994) and the BIA-d (Grainger et al., 2010) even though the rationale and mechanisms implicated are not the same. Both theoretical accounts suggest that at initial stages of L2 learning the lexico-semantic organization of L2 words are mostly mediated by L1 translations, and therefore the process of acquiring new L2 vocabulary is highly sensitive to the overlap at the orthographic and/or phonological and semantic levels with the L1. In contrast, strong mappings between L2 lexical representations and concepts are needed in order to achieve good reading comprehension at intermediate levels of proficiency. Therefore, at intermediate levels of proficiency the degree of reliance on cross-language similarity is inversely related with reading comprehension achievement, suggesting that those L2 learners who at the end of the first academic term presented a decrease on the strength of L1 reliance in favor of the direct links between L2 lexical representations and semantic concepts (as measured by a decrease in the cognate effect) had better prognostic at achieving good reading comprehension scores at the end of the academic year.

These results also demonstrate a close relationship between general cognitive skills and individual differences in (non-native) language learning. The estimate of non-verbal intelligence significantly explained part of the variance of the subsequent reading language comprehension in both groups (Experiments 1 and 2), in line with preceding studies (Papagno and Vallar, 1995; Kempe et al., 2010; Lopez-Barroso et al., 2011). Higher non-verbal intelligence scores provided a better prognostic of reading comprehension. It is well known that fluid intelligence is closely related with executive functions (Carpenter et al., 1990; Miyake et al., 2001; Salthouse et al., 2003; Colom et al., 2006) and reasoning abilities (Duncan et al., 1995, 1996). Furthermore, IQ has been found to be a good predictor of general academic performance (Murray and Lamb, 1994; Gottfredson, 2004; Coyle and Pillow, 2008; Herrnstein and Murray, 2010). Our results add to a growing body of evidence showing a link between non-verbal intelligence and academic performance in a non-native language-learning context (see Pishghadam and Khajavy, 2013).

Similarly, and in line with preceding evidence, results from Experiment 2 showed that WM capacity is also related to non-native language achievement, at least at intermediate levels of proficiency. It has been previously shown that WM capacity is highly correlated not just with general reading comprehension, but with general reasoning abilities (Daneman and Carpenter, 1980; Turner and Engle, 1989; Kyllonen and Christal, 1990), mathematical processing (Ashcraft, 1995; Gathercole and Pickering, 2000; Lee and Kang, 2002; Kyttälä and Lehto, 2008) and attentional control, among other cognitive skills (Barrett et al., 2004; Wright et al., 2014). In that sense, WM capacity was also positively related with good reading achievement at intermediate levels of proficiency, but not at lower levels, suggesting that this factor could play a different role at different stages of language learning. In general, it seems that both factors (IQ and WM) contribute significantly to the general learning processes, and it is not surprising that similar effects are also found in experimental contexts aimed at exploring non-native language learning processes.

One question that remains open is whether the two groups differ in their general cognitive abilities. Kroll et al. (2002) investigated the role of WM (as measured with an L1 reading span task) at different levels of L2 achievement in a series of L1/L2 word naming and explicit translation tasks. They found that L2 learners displayed a generally lower reading span than highly proficient bilinguals. Interestingly, we found a similar pattern of results. The more proficient group had a greater memory span than the low proficient group [*t*(165) = 2.81; *p* = 0.005]. In addition, the more proficient learners also had a significantly higher IQ [*t*(165) = 2.48; *p* = 0.01], even though we had no expectation that general cognitive factors would differ between participants at different levels of proficiency. Certainly, deciphering whether these differences are a consequence or a cause of non-native language proficiency in the present study is not possible, and it goes beyond the scope of this manuscript. Nonetheless, we believe that this is a suggestive finding that is worth mentioning.

More directly related with the main goal of this study, Kroll et al. (2002) showed that the cognate effect was inversely related with WM scores. They showed that at lower levels of proficiency, the group with higher memory span showed reduced cognate effects compared to the group with lower memory span. In partial contrast with these results, we found that the performance in the WM and intelligence tests were not related to the cognate effect. Even though there was a marginal positive relationship between the magnitudes of the cognate effect and IQ estimates in the A2 group (*r* = 0.23; *p* = 0.07), such a relationship was not found for the WM estimates (*r* = 0.05; *p* = 0.69). For the more proficient B1 group none of these estimates showed a significant correlation with the cognate effect (IQ: *r* = -0.15; *p* = 0.14; WM: *r* = 0.08; *p* = 0.45). Nonetheless, it should be kept in mind that the reading span task used by Kroll et al. (2002) is a more complex task than the forward number retention task used in the current study to estimate WM (see Daneman and Carpenter, 1980, for a direct comparison between both tasks and different reading tests). In addition, it should be noted that the translation naming task used by Kroll et al. (2002) require a deeper lexical search and an involvement of WM should probably be more anticipated in that task than in the lexical decision task used in the current study.

Interestingly, our results also showed that the chronological age of participants (which varied from 19 to 69 years) did not account for individual differences in reading comprehension achievement in either group. In contrast to the common view that non-native language achievement is hampered by age, these results demonstrate that the non-native second language acquisition age is more relevant for language attainment than the actual age of the learners (see Dowens et al., 2010, for a similar conclusion) at intermediate levels of proficiency. Furthermore, while these two variables show a high degree of cross-correlation due to the relatively recent structural changes in the educational policies in Spain leading to the inclusion of English in the formal academic curriculum, chronological age, but not age of English acquisition, was dropped from both models resulting in a negligible change in the predictive capacity of the general regression models.

Previous studies have shown that learning a foreign language is positively influenced by previous experience with other languages (Sanz, 2000; Cenoz, 2003, 2013; Errasti, 2003; Pilar and Jordà, 2003; Safont, 2005; Kassaian and Esmae’li, 2011; Zare and Mobarakeh, 2013; among others). All participants tested in our study were native Spanish speakers living in a bilingual region (the Basque Country). Hence, all of them had some knowledge of Basque, the co-official language in this region (self-ratings of Basque proficiency of the participants in A1 level according to a 1-to-10 scale: mean = 5.53, SD = 2.84; level B1: mean = 6.52, SD = 2.58). To investigate the potential impact of knowledge and exposure to Basque on English learning, we reanalyzed the models by adding the participants’ self-ratings of Basque proficiency as a factor. The inclusion of this factor did not significantly improved the predictability of second language achievement for the low proficiency group [Adjusted *R*^2^ = 0.23; *F*-change(1,57) = 0.63; *p* = 0.43]. In other words, the variability associated with the prior knowledge of another language did not modulate foreign language comprehension achievement at lower levels of formal lessons [βs = 0.09, *t*(61) = 0.79, *p* > 0.40]. However, inclusion of Basque proficiency into the model improved the predictability of English comprehension achievement of intermediate learners of English at the end of the scholar year, even though the effect was marginal [Adjusted *R*^2^ = 0.21; *F*-change(1,99) = 3.68; *p* = 0.06]. Participants with higher knowledge of Basque showed a marginal advantage at achieving better scores in English reading comprehension [βs = 0.19, *t*(104) = 1.92, *p* = 0.06]. This suggests that knowing another language different from the native language facilitates learning a foreign language, even though this positive impact of bilingualism only appeared for intermediate levels of language acquisition and was not very robust.

Overall, the results suggest that the cognate facilitation effect, a purely psycholinguistic measure that has been found to be a good predictor of word identification across different language combinations (Lemhöfer et al., 2008) and that is highly influenced by the degree of proficiency in the non-native language (Bultena et al., 2014), predicts reading comprehension skills in non-native language learners at the end of the academic year. More specifically, our results indicate that higher achievement in reading comprehension is predicted by lower cognate facilitation effects assessed several months before the exam at intermediate levels of proficiency (Experiment 2), and that higher cognate effects predict higher reading comprehension achievement at initial levels of foreign language learning (Experiment 1). These findings fit well with previous evidence from studies testing bilingual reading comprehension showing an inverse relationship between the magnitude of the cognate effect and the level of proficiency in a given non-native language. The cognate effect was a good predictor of reading comprehension achievement at the end of the school year, showing a positive relationship between cognate effects and reading comprehension at lower levels of proficiency, and an inverse correlation between cognate effects and reading comprehension at intermediate levels of proficiency (see **Figures 1B and 2B**). As discussed above, at the theoretical level these results fit well with models suggesting that during the course of non-native language learning, bilinguals move from a non-native word recognition process that mainly relies on the pre-existing native-language representations (the L1-lexical mediation hypothesis), to a direct conceptual access that is mainly dependent on the non-native linguistic representations (Potter et al., 1984; De Groot and Nas, 1991; De Groot, 1992; De Groot et al., 1994; Kroll and Stewart, 1994; Kroll and De Groot, 1997; Perea et al., 2008; Kroll et al., 2010). The current results confirm these hypotheses and further demonstrate that a greater reliance on native-language lexical mediation strategies goes hand in hand with impoverished non-native reading comprehension skills at intermediate levels of proficiency.

To conclude, these results suggest that individual differences in non-native reading comprehension achievement are highly influenced by general cognitive and linguistic factors, and critically, by the extent to which learners rely on their native language during the non-native language learning process.

## Conflict of Interest Statement

The authors declare that the research was conducted in the absence of any commercial or financial relationships that could be construed as a potential conflict of interest.

## Footnotes

1 Corrected Levensthein distance was used to calculate the cognate status of words between English and Spanish, ranging from 0 (non-cognates) to 1 (fully cognates).

2 http://www.coe.int/t/dg4/linguistic/cadre1_en.asp

3 In general, the cognate effect was larger for the low proficient group (mean RT effect = 61; SD = 80; mean accuracy effect = 7.84%, SD = 9.67) than for the intermediate proficient group (mean RT effect = 47; SD = 48; mean accuracy effect = 5.47%, SD = 5.44). A combined analysis on the latency and accuracy data including the factor Group showed that the cognate effect was larger in the group with lower proficiency. This difference was statistically significant in the analysis on the accuracy data [*F*1(1,165) = 4.13, *p* = 0.04; *F*2(1,198) = 3.63, *p* = 0.05], and marginal in the RT analysis, in which only the by-item analysis showed a significant difference [*F*1(1,165) = 1.84, *p* = 0.17; *F*2(1,198) = 13.86, *p* < 0.001].
