# Supplementary material for: Foreign language comprehension achievement: insights from the cognate facilitation effect
Source: Front Psychol. 2015 May 6;6:588. doi: 10.3389/fpsyg.2015.00588 (PMC4421941; doi:10.3389/fpsyg.2015.00588)
Supplement: Supplementary file 1 [file Data_Sheet_1.PDf]

Supplementary Material

Foreign language comprehension achievement: insights from the cognate facilitation effect

Aina Casaponsa*; Eneko Antón; Alejandro Pérez; Jon Andoni Duñabeitia

Correspondence: Aina Casaponsa, Basque Center on Cognition, Brain and Language, Paseo Mikeletegi 69 2nd, 20009 Donostia, Spain
a.casaponsa@bcbl.eu

1. Supplementary Material.

Materials used in the lexical decision task (Spanish translations are provided between brackets).

COGNATES: factor (factor); ideal (ideal); paper (papel); invention (invención); athlete (atleta); domination (dominación); literature (literatura); minister (ministro); tourist (turista); optimism (optimismo); dimension (dimensión); hospital (hospital); monarch (monarca); function (función); desert (desierto); metal (metal); system (sistema); observer (observador); secretary (secretario); palace (palacio); tone (tono); mineral (mineral); volume (volumen); ruin (ruina); crime (crimen); prison (prisión); tobacco (tabaco); author (autor); industry (industria); sperm (esperma); pole (polo); actor (actor); material (material); statue (estatua); direction (dirección); prosperity (prosperidad); guitar (guitarra); prudent (prudente); education (educación); abundance (abundancia); diet (dieta); service (servicio); complex (complejo); fame (fama); calm (calma); minute (minuto); control (control); crisis (crisis); continent (continente); culture (cultura); convent (convento); cause (causa); bronze (bronce); procession (procesión); instinct (instinto); solution (solución); million (millón); prose (prosa); curve (curva); legion (legión); arch (arco); monument (monumento); torture (tortura); hotel (hotel); history (historia); distance (distancia); cement (cemento); pulse (pulso); reaction (reacción); curious (curioso); angel (ángel); expedition (expedición); abdomen (abdomen); list (lista); calcium (calcio); problem (problema); audience (audiencia); emperor (emperador); sex (sexo); segment (segmento); chaos (caos); crystal (cristal); discussion (discusión); candidate (candidato); conflict (conflicto); remedy (remedio); detail (detalle); pistol (pistola); silence (silencio); valley (valle); accident (accidente); tradition (tradición); colonel (coronel); insect (insecto); capital (capital); fiction (ficción); canal (canal); idea (idea); clan (clan); ceremony (ceremonia).

NON-COGNATES: shoulder (hombro); sickness (mareo); opening (apertura); lazy (vago); business (negocio); length (duración); feeling (sentimiento); removal (traslado); wisdom (sabiduría); sword (espada); spring (primavera); load (carga); lounge (sala); singer (cantante); bitterness (amargura); ribbon (cinta); wealth (riqueza); belief (creencia); revenge (venganza); challenge (desafío); weakness (debilidad); advice (consejo); teacher (profesor); needle (aguja); beauty (belleza); spark (chispa); expense (gasto); goodness

(bondad); beginning (principio); madness (locura); council (ayuntamiento); denial (negativa); management (gestión); success (éxito); childhood (infancia); kingdom (reino); thread (hilo); exchange (intercambio); lack (falta); attempt (intento); leisure (ocio); amount (cuantía); lieutenant (teniente); slope (cuesta); fight (pelea); grass (hierba); crowd (muchedumbre); threat (amenaza); matter (tema); curse (maldición); witness (testigo); warehouse (almacén); building (edificio); deity (divinidad); shower (ducha); thigh (muslo); parade (desfile); twilight (crepúsculo); church (iglesia); rabbit (conejo); newspaper (periódico); youth (juventud); whiteness (blancura); thief (ladrón); whisper (susurro); freedom (libertad); afternoon (tarde); safety (seguridad); bunch (ramo); silver (plata); shortage (escasez); century (siglo); driver (conductor); darkness (oscuridad); shepherd (pastor); harvest (cosecha); polite (cortés); village (pueblo); speech (habla); drought (sequía); customer (cliente); fright (susto); kindness (amabilidad); smell (olor); flight (vuelo); fullness (plenitud); prayer (oración); pity (pena); teacher (maestro); summer (verano); umbrella (paraguas); chess (ajedrez); welcome (bienvenida); device (dispositivo); strength (fuerza); mirror (espejo); knowledge (conocimiento); balance (equilibrio); mood (humor); season (temporada).

NONWORDS: ranute; sunailment; runarch; lood; conflart; afterceed; lebean; squead; reeysion; wistpaper; atcemen; burterners; ramution; chirtwood; sareral; pobbit; prunon; trallemns; prote; palness; indombly; beeching; asuncence; frief; trablem; exglinge; catciog; pirter; pollion; doldness; cran; bunce; cutfure; bernness; shupent; thisk; adagition; welmime; optisals; wamehound; advigent; basades; conpant; kingtum; complag; allempt; arelene; nooble; mader; foucher; matedy; wadness; grocition; speedam; ingell; taky; icoac; maller; midibation; cunge; tove; slirer; pulfs; peciac; dencossion; mimmer; sucension; pery; hencory; shring; fape; slode; detive; squeat; sarument; palode; ciciees; spight; ansal; smitight; cortnet; chulks; cebant; ulshulla; mopegiad; molver; spyntal; potind; distides; asient; cacel; hanrest; holet; boogty; muid; shamer; tex; weanse; oblailer; swateness; tenction; adhace; prorarsion; sheyhird; myntem; loaling; cucts; resange; cact; twell; tursure; lourse; sarister; croif; wrodmeraty; cettower; sotal; oculing; alks; hooftenant; polmetary; fiffs; lirtion; kertness; conglil; grale; poss; shintage; jallem; ylphs; pentol; esdense; caire; beroof; itceror; swork; lims; miccess; spaus; drisder; catitot; vellays; chitue; rirton; pilette; lesotes; ercomition; slark; dicontion; meabon; bronde; lellness; fawbor; moud; indlisks; devole; ahriered; spouch; encontion; naipy; crilt; lans; cimagony; blought; fobbital; knornepts; tobilpo; rackness; siel; befelning; teatist; praire; bribis; coungyl; catcinent; weedness; pasave; middor; bederatums; cheelder; sensile; streytes; awbor; surkty; ucoa; foucher; demelt; cunducy; vusume; wisbim; candibent; snight; seodent; geepness; awplor; liecure; spefs; shess; gukbar; recigal.

2. Supplementary Figures.

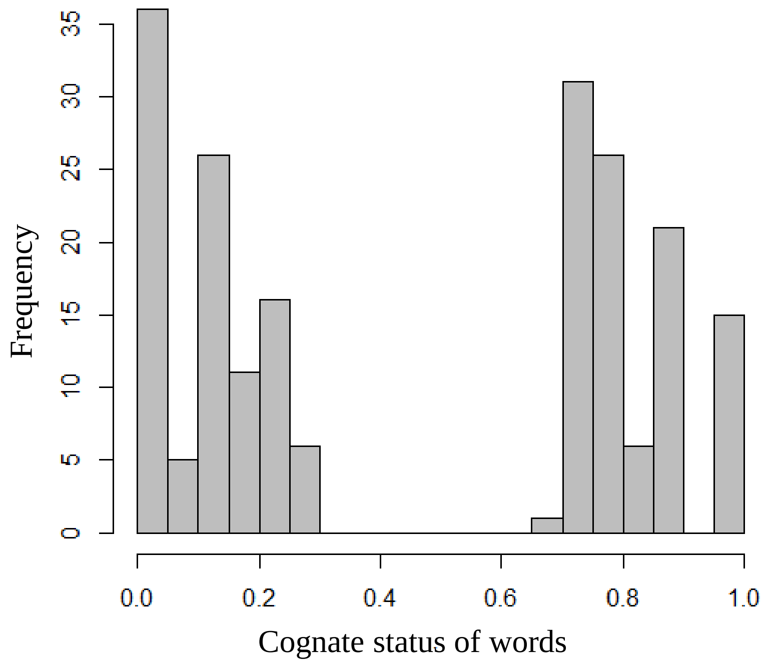

Supplementary Figure 1. Distribution of the cognates and non-cognates words along the continuum of cognate status calculated with length-corrected Levenshtein distance (0 indicates no overlap and 1 indicates fully cognates).
